# Stepwise bioprocess for exopolysaccharide production using potato starch as carbon source

**DOI:** 10.1007/s13205-014-0273-2

**Published:** 2014-12-23

**Authors:** Shashi Kant Bhatia, Narinder Kumar, Ravi Kant Bhatia

**Affiliations:** 1 Department of Biotechnology, Indian Institute of Education, Ghanahatti, Shimla, 171011 India; 2 Department of Biotechnology, Himachal Pradesh University, Shimla, 171005 India

**Keywords:** Biopolymer, Biosynthesis, Downstreaming process, Xanthan gum

## Abstract

Xanthan gum is a biopolymer produced by *Xanthomonas* sp. XC6. In this study, xanthan gum is produced from potato starch using a stepwise bioprocess design. Potato starch is hydrolyzed using *Bacillus* sp. having amylase activity and 30.2 g/L reducing sugar was released, while *Xanthomonas* sp. XC6 can release only 14.5 g/L. *Bacillus* sp. hydrolyzed potato starch extract was further used as a carbon source for xanthan gum biosynthesis using *Xanthomonas* sp. XC6. Yeast extract acts as the best nitrogen source, and 10.0 g/L xanthan gum was recovered. Downstreaming process after stepwise bioprocess resulted in 17.4 g/L xanthan gum production, which is 2.8 times higher as compared to single step process.

**Electronic supplementary material:**

The online version of this article (doi:10.1007/s13205-014-0273-2) contains supplementary material, which is available to authorized users.

## Introduction

Biopolymer is a type of polymer which is produced by living organisms; it contains monomeric units which are covalently bonded to form larger structures (Nwodo et al. 2012). The exopolysaccharide (EPS) is a biopolymer which is composed of high molecular weight polymers made up of sugar residues, and responsible for the architecture and morphology of the matrix in which cells live and serve as a potential energy reserve (Sayyed et al. 2012). Exopolysaccharides of microbial origin have immense potential to be used in food and pharma industry (Gong et al. 2009; Fanga et al. 2013). Xanthan gum is the extracellular polysaccharide produced by *Xanthomonas* sp. and commercially accepted (Ghazal et al. 2011; Oliveira et al. 2013). It is a heteropolysaccharide and consists of glucose, mannose, and glucuronic in 2:2:1 proportion (Gilani et al. 2011). Bacterium xanthan gum is an attractive alternative for the replacement of traditional gums obtained from plants and marine algae by chemical extraction process (Freitas et al. 2011). Because of its special rheological properties, xanthan gum is used in pharmaceuticals, paper, paint, food, cosmetics, and textile industries due to its high viscosity at low concentrations; pseudo plasticity; and insensitivity to a wide range of temperature and pH (Gilbert et al. 2013; Patel and Prajapati 2013). In large-scale production of xanthan gum, glucose is considered as a suitable substrate with high production and yield (Zhang and Chen 2010). However, the increasing market demand suggests that glucose may no longer be economically feasible as a raw material, and in order to reduce the costs of raw material for xanthan gum production low cost carbon sources were recommended. There are a number of research studies devoted to identifying a suitable substrate with low costs (Mudoi et al. 2013). Several methods have been developed to increase the yield and properties of xanthan gum using various carbon sources (Silva et al. 2009). The objective of this work is to increase the production of xanthan gum and to find an alternate of glucose as carbon source. In this study, *Bacillus* sp. is used in the saccharification of starch as it is a well established organism and has been used to develop a number of bioprocesses (Patel et al. 2012; Sangkharak and Prasertsan 2013). *Bacillus* sp. is regarded generally as safe (GRAS) by the Food and Drug Administration. Fermented broth obtained after starch hydrolysis by *Bacillus* sp. was further used as a carbon source for xanthan gum production using *Xanthomonas* sp. XC6.

## Materials and methods

### Chemicals

All the media components were obtained from Hi-Media (Mumbai, India) and other chemicals were procured from Merck (India).

### Microorganism and culture conditions

The infected fruit and leaf from citrus plants showing canker symptom were collected from Shimla, Himachal Pradesh for the isolation of *Xanthomonas* sp. Isolation was carried out in glucose/yeast/calcium carbonate agar (GYCA) medium (Freitas et al. 2011). The necrotic lesions from leaves and fruits were dissected and surface sterilized in 70 % alcohol. The infected samples were then suspended in a small test tube containing 3 mL saline and incubated at 30 °C for 24 h, after that the bacterial suspension were serially diluted in 9 mL sterile distilled water. Then 0.1 mL of the diluted bacterial cell suspension was poured into sterilized petri plates containing GYCA medium and incubated for 30 °C for 72 h. Wild type isolates were selected by choosing yellow, viscous, and convex colony having a diameter of 4 mm. Isolated strains were screened for their xanthan gum production potential. Selected strain was identified by using phenotypic and biochemical characteristics, i.e. morphology, motility, starch, gelatin hydrolysis, and carbohydrate utilization (glucose, fructose, sucrose, maltose, and glycerol) tests. For 16 s rRNA analysis, genomic DNA was isolated from the bacterial isolates and used as template for PCR. PCR was performed using MycyclerTM (Bio-Rad, USA). The sequence data was checked by BLAST analysis and phylogentic tree was constructed using NCBI (phylip programme). Other strain *Bacillus* sp. was received from Microbial Type Culture Collection and Gene Bank (IMTECH).

### Xanthan gum estimation

Biomass and xanthan gum estimation were performed after withdrawing the sample, for biomass estimation 5 mL sample was centrifuged at 12,000 rpm for 10 min, then supernatant is decanted and cell pellet is dried in hot air oven at 100 °C and then weighed. Xanthan gum was extracted from the supernatant using 95 % ethanol for precipitation. The precipitates were separated by centrifugation at 10,000 rpm for 15 min and dried at 60 °C and weighed (Meng et al. 2010).

### Optimization of cultural conditions

#### Carbon source

Different carbon (starch, maltose, glucose, and sucrose) sources were tested for biomass and xanthan gum production by *Xanthomonas* sp. XC6 at 30 °C for 120 h. Samples were withdrawn at 24 h intervals and analyzed for xanthan gum production.

#### Starch hydrolysis and saccharification

Isolated bacteria *Xanthomonas* sp. XC6 can utilize maltose as a carbon source for biomass and xanthan gum production, so potato starch can be used as a cheaper substrate for xanthan gum production. Potato tubers were washed with distilled water to remove dirt, peeled manually and boiled. Boiled potatoes were crushed, mixed with adequate amount of water, filtered through a Teflon cloth, and autoclaved at 121 °C. Saccharification of the potato starch extract was carried out using *Xanthomonas* sp. XC6 and *Bacillus sp.* Potato extract containing g/L, Na_2_HPO_4_·12H_2_O, 2.5 g; K_2_HPO_4_, 2.0 g; MgSO_4_·7H_2_O, 1.0 g; FeSO_4_·7H_2_O, 0.1 g; and CaCl_2_·2H_2_O, 0.6 and yeast extract 5.0 g was used as medium. *Xanthomonas* sp. and *Bacillus* sp. preculture were inoculated separately in 50 ml medium containing in Erlenmeyer conical flask, and saccharification was carried out at 37 °C for 96 h. Samples were withdrawn and analyzed for total reducing sugar release using the DNS method (Rajesh et al. 2013).

#### Xanthan gum production using Xanthomonas sp

*Bacillus* sp. scarified potato extract was centrifuged at 15,000 rpm for 15 min to remove the cell and further subjected for xanthan gum production. The hydrolyzed potato starch extract was used as carbon source and inoculated with the preculture of *Xanthomonas* sp. and incubated at 37 °C and further cultural condition optimization was performed.

#### Effect of nitrogen source and temperature

Fermented broth obtained after hydrolysis of potato starch by *Bacillus* sp. was used as sole carbon source, and various commercially available nitrogen sources (peptone, yeast extract, beef extract, and malt extract at 1 %) were checked out to increase the production of xanthan gum. Effect of temperature was also studied by varying it from 20 to 45 °C. After optimization of cultural condition, xanthan gum production at 1 L performed using fermented broth of *Bacillus* sp. and reducing sugar content profile was studied under optimized condition up to 120 h.

#### Xanthan gum production at bench scale

Under the optimized cultural condition, stepwise fermentation (SF) and simultaneous saccharification and fermentation study were performed at 1L scale. In stepwise fermentation, *Bacillus* sp. was used to hydrolyze potato starch extract and saccharified broth containing 30.2 g/L reducing sugar was further incubated at 30 °C for 72 h under shaking condition with *Xanthomonas sp.* XC6 for Xanthan gum production. In simultaneous saccharification and fermentation (SSF), inoculation of potato extract containing optimized medium components directly with preculture of *Xanthomonas* sp. XC6 was also performed for xanthan gum production under optimized condition. Exopolysaccharide extraction was performed and FTIR analysis was done.

### Fourier-transformed infrared spectroscopy (FTIR)

Xanthan gum was extracted from fermented broth as mentioned above. Samples of purified EPS were prepared for FTIR analysis. One mg of the purified EPS was used in analysis by using salt discs. A mixture made by adding 1 mg of EPS samples to 300 mg of pure dried KBr followed by pressing into the disc, the whole FTIR spectrum was analyzed at 400–4,200 cm^−1^ using Shimadzu FTIR 8300 Spectrophotometer (Shimadzu, Tokyo, Japan).

## Results

### Isolation

**Fig. 1:**
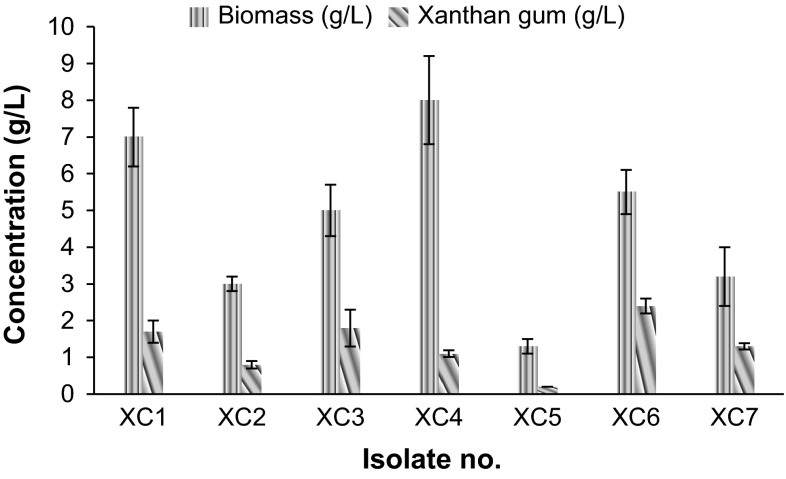
Xanthan gum production by different isolated bacterial strains

Totally seven isolates were isolated using GYCA medium and characterized for xanthan gum production. Isolate XC6 showed maximum (2.4 g/L) xanthan gum production with 5.5 g/L biomass production. Bacterial isolate XC1 and XC4 showed good biomass production, but low production of xanthan gum was recorded. Isolates XC2, XC5 and XC7 showed little biomass production as well as xanthan gum production (Fig. 1). Bacterial isolate having higher xanthan gum production potential is identified as *Xanthomonas* sp. XC6 on the basis of biochemical tests (Table. S1) and confirmed by performing 16 s rRNA (Figure S1). 16 s rRNA sequence showed maximum homology (99 %) with *Xanthomonas campestris*.

### Optimization of carbon source

**Fig. 2:**
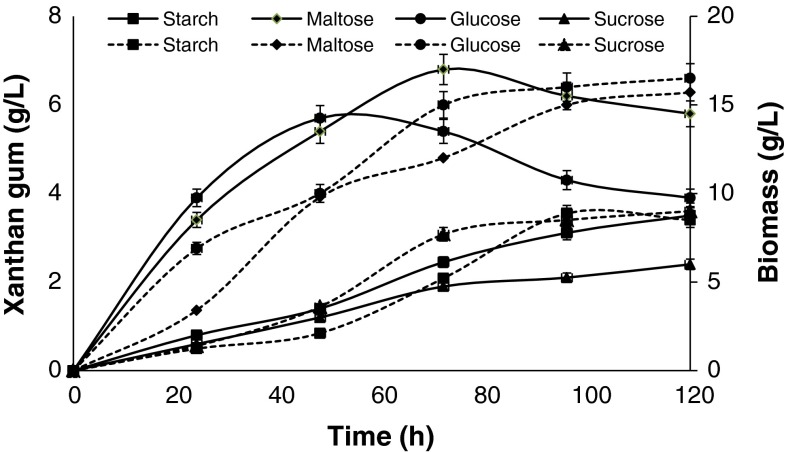
Xanthan gum production and growth profile of *Xanthomonas* sp. XC6 on different carbon source

Out of four commercially available carbon source used for xanthan gum production, maltose proved best carbon source for xanthan gum (6.8 g/L) production (Fig. 2). In glucose, *Xanthomonas* showed good biomass production as compared to maltose but xanthan gum production (5.7 g/L) was low (Fig. 2). Xanthan gum production increased with biomass production and the optimum time for maximum xanthan gum was recorded as 72 h in maltose as carbon source. Starch is the second most abundantly available carbon source and can be scarified into maltose using bacterial fermentation. *Bacillus* sp. and *Xanthomonas* sp. were used separately for the saccharification of potato starch extract to release the reducing sugar. *Bacillus* sp. released 30.2 g/L reducing sugar in 48 h while *Xanthomonas* sp. released 14.5 g/L after 72 h.

### Nitrogen source and temperature

**Fig. 3:**
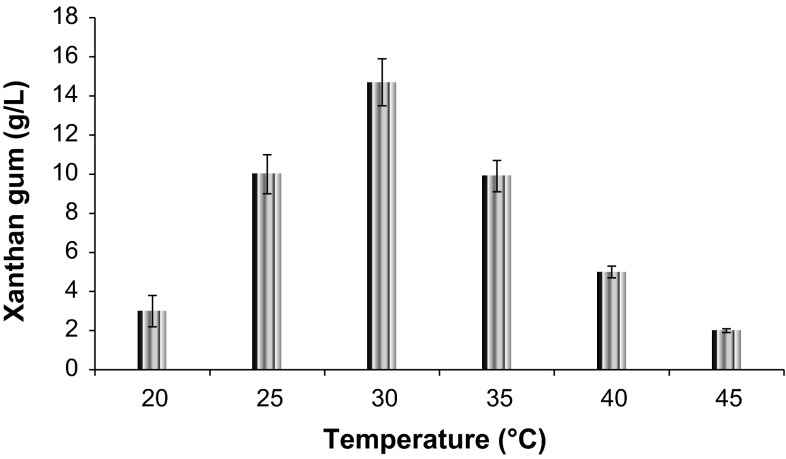
Effect of temperature on xanthan gum production by *Xanthomonas* sp. XC6

**Fig. 4:**
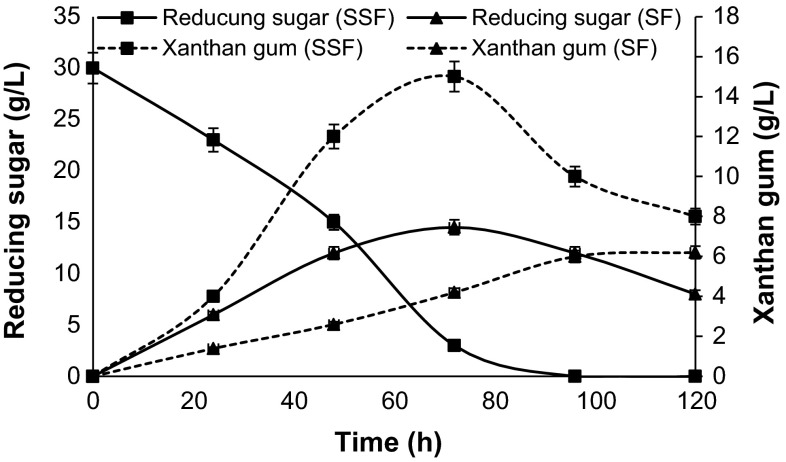
Xanthan gum production and reducing sugar profile during stepwise and simultaneous saccharification and fermentation

*Xanthomonas* sp. yields 10.0 g/L xanthan gum in 72 h when yeast extract is used as nitrogen source (Figure S2). Peptone also showed good xanthan gum production, but less in comparison to yeast extract. Beef extract and malt extract act as poor nitrogen source. With the increase of temperature increase in xanthan gum production (14.7 g/L) was recorded up to 30 °C (Fig. 3). At 1 L scale, xanthan gum production and reducing sugar profile were studied, and 90 % reduction in sugar content was recorded at 72 h (Fig. 4) in stepwise fermentation. In SSF process, reducing sugar and xanthan gum production go on simultaneously, and maximum reducing sugar of about 14.5 g/L is released in 72 h. SSF at 1 L scale can yield only 6.2 g/L xanthan gum, while stepwise approach under optimized condition result in 17.4 g/L xanthan gum (Fig. 4).

### FTIR analysis

Fourier-transformed infrared spectroscopy of the xanthan gum produced from the *Xanthomonas* sp. using stepwise fermentation processes was performed. The *x*-axis represents wavelength (cm^−1^) and *y*-axis shows the light transmittance through the sample. Many functional groups were observed: a stretch around 3,464 cm^−1^ shows the presence of –OH group, peak between 2,880 and 2,967 cm^−1^ represents –CH stretching of methyl groups, peak at 1,746 represents the stretching of –C=O group, strong signals at 1,746 show the presence of –C=O stretching of the acetate group, and a peak at 1,455 shows the presence of –COOH group.

## Discussion

Xanthan gum is the most commercially produced industrial gum, obtained by fermentation. Development of high-performance xanthan gum-producing microorganisms, reduction of the costs of raw materials, and improvement of fermentation processes are the different approaches that can be used to make production process effective. In this study, xanthan gum-producing bacterial culture *Xanthomonas* sp. XC6 was isolated from citrus plants having canker disease symptoms and further used for xanthan gum production using starch as substrate. This work showed the importance of the stepwise process for xanthan gum production, as starch is a cheap carbon source and saccharification using amylase activity of *Bacillus* sp. resulted in fermentation broth with higher sugar content. *Bacillus* sp. released 30.2 g/L reducing sugar in 48 h while *Xanthomonas* sp. XC6 released 14.5 g/L after 72 h. So a stepwise process, saccharification of potato starch with *Bacillus* sp. and xanthan gum production using *Xanthomonas* sp,. was used. Previously, different research group has reported the production of xanthan gum using different materials (whey, molasses, and cassava starch) as carbon source (Kerdsup et al. 2011; Mudoi et al. 2013; Oliveira et al. 2013; Silva et al. 2009). Nitrogen source has an important role in biomass production as well as xanthan gum production. Yeast extract acts as a best nitrogen source for xanthan gum production for *Xanthomonas* sp. XC6 as already reported for xanthan gum production by *Xanthomonas campestries* (Gilbert et al. 2013). Temperature also plays a critical role in xanthan gum production, and 30 °C is the optimum temperature for maximum xanthan gum production in *Xanthomonas* sp. XC6. The influence of temperature on xanthan gum production has been widely studied by different research groups and found that 28 °C was the optimal temperature for xanthan gum production (Gumus et al. 2010; Kerdsup et al. 2011; Silva et al. 2009). Under the optimized cultural condition, single step process (SSF) at 1 L scale can yield 6.2 g/L xanthan gum. Xanthan gum production with a stepwise approach at 1 L scale, under optimized conditions, resulted in 17.4 g/L, while 5.97 g/L xanthan gum production was reported in *Xanthomonas campestris* TISTR 840 using cassava starch as carbon source (Silva et al. 2009). Fermentation using *Xanthomonas* *campestris* on waste residual molasses produces only 12.23 g/L xanthan gum (Murugesan et al. 2012). Fourier-transformed infrared spectroscopy of xanthan gum, extracted after stepwise process, was performed and it gives the same type of FTIR spectra, as obtained for xanthan gum produced from *Xanthomonas campestris* using waste residual molasses (Mudoi et al. 2013).

The potentiality of this stepwise approach for xanthan gum production from starch using *Xanthomonas* sp. XC6 seems to be advantageous over single step process (SSF). Stepwise bioprocess resulted in a 64.36 % increase in xanthan gum production as compared to single step approach under optimized condition.

## Electronic supplementary material

Below is the link to the electronic supplementary material.

Supplementary material 1 (DOCX 281 kb)

## References

[CR1] Fanga Y, Ahmed S, Liua S, Wanga S, Lua M, Jiaoa Y (2013). Optimization of antioxidant exopolysaccharidess production by *Bacillus licheniformis* in solid state fermentation. Carbohydr Polym.

[CR2] Freitas F, Alves VD, Reis MAM (2011). Advances in bacterial exopolysaccharides: from production to biotechnological applications. Trends Biotechnol.

[CR3] Ghazal SMA, Elsayed WS, Badr UM, Gebreel HM, Khali KMA (2011). Genetically modified strains of *Xanthomonas campestris* higher xanthan producer and capable to utilize whey. Curr Res Bacteriol.

[CR4] Gilani SL, Najafpou GD, Heydarzadeh HD, Zare H (2011). Kinetic models for xanthan gum production using *Xanthomonas campestris* from molasses. Chem Ind Chem Eng.

[CR5] Gilbert L, Loisel V, Savary G, Grisel M, Picard C (2013). Stretching properties of xanthan, carob, modified guar and celluloses in cosmetic emulsions. Carbohydr Polym.

[CR6] Gong Y, Wang C, Lai RC, Su K, Zhang F, Wang D (2009). An improved injectable polysaccharide hydrogel: modified gellan gum for long-term cartilage regeneration in vitro. J Mater Chem.

[CR7] Gumus T, Demirci AS, Mirik M, Arici M, Aysan Y (2010). Xanthan gum production of *Xanthomonas* spp. isolated from different plants. Food Sci Biotechnol.

[CR8] Kerdsup P, Tantratian S, Sanguandeekul R, Imjongjirak C (2011). Xanthan production by mutant strain of *Xanthomonas campestris* TISTR 840 in raw cassava starch medium. Food Bioprocess Technol.

[CR9] Meng F, Zhou B, Lin R, Jia L, Liu X, Deng P, Fan K, Wang G, Wang L, Zhang J (2010). Extraction optimization and in vivo antioxidant activities of exopolysaccharide by *Morchella esculenta* SO-01. Bioresour Technol.

[CR10] Mudoi P, Bharali P, Konwar BK (2013). Study on the Effect of pH, Temperature and aeration on the cellular growth and xanthan production by *Xanthomonas campestris* using waste residual molasses. J Bioprocess Biotech.

[CR11] Murugesan AG, Dhevahi B, Gowdhaman D, Bala AK, Sathesh PC (2012). Production of xanthan employing *Xanthomonas campestris* using sugarcane molasses. Am J Environ Eng.

[CR12] Nwodo UU, Green E, Okoh AI (2012). Bacterial Exopolysaccharides: functionality and Prospects. Int J Mol Sci.

[CR13] Oliveira PD, Michel RC, McBride AJA, Moreira AS, Lomba RFT, Vendruscolo CT (2013). Concentration regimes of biopolymers xanthan, tara, and clairana, comparing dynamic light scattering and distribution of relaxation time. PLoS One.

[CR14] Patel P, Prajapati JB (2013). Food and health applications of exopolysaccharides produced by lactic acid bacteria. Adv Dairy Res.

[CR15] Patel SKS, Singh M, Kumar P, Purohit HJ, Kalia VC (2012). Exploitation of defined bacterial cultures for production of hydrogen and polyhydroxybutyrate from pea-shells. Biomass Bioenerg.

[CR16] Rajesh T, Kim YH, Choi YK, Jeon JM, Kim HJ, Park SH, Park HY, Choi KY, Kim H, Kim HJ, Lee SH, Yang YH (2013). Identification and functional characterization of an α-amylase with broad temperature and pH stability from *Paenibacillus* sp. Appl Biochem Biotechnol.

[CR17] Sangkharak K, Prasertsan P (2013). The Production of Polyhydroxyalkanoate by *Bacillus licheniformis* using sequential mutagenesis and optimization. Biotechnol Bioprocess Eng.

[CR18] Sayyed RZ, Jamadar DD, Patel PR (2012). Production of Exo-polysaccharide by *Rhizobium* sp. Indian J Microbiol.

[CR19] Silva MF, Fornari RCG, Mazutti MA, Oliveira D, Padilha FF, Cichoski AJC, Cansian RL, Luccio MD, Treichel H (2009). Production and characterization of xantham gum by *Xanthomonas campestris* using cheese whey as sole carbon source. J Food Eng.

[CR20] Zhang Z, Chen H (2010). Fermentation performance and structure characteristics of xanthan produced by *Xanthomonas campestris* with a glucose/xylose mixture. Appl Biochem Biotechnol.

