# Supplementary material for: Stepwise bioprocess for exopolysaccharide production using potato starch as carbon source
Source: 3 Biotech. 2014 Dec 23;5(5):735–9. doi: 10.1007/s13205-014-0273-2 (PMC4569638; doi:10.1007/s13205-014-0273-2)
Supplement: Supplementary file 1 — Supplementary material 1 (DOCX 281 kb) [file 13205_2014_273_MOESM1_ESM.docx]

**Figure S1 16s rRNA analysis and phulyogenetic tree of xanthan gum producing bacterial isolate**

**
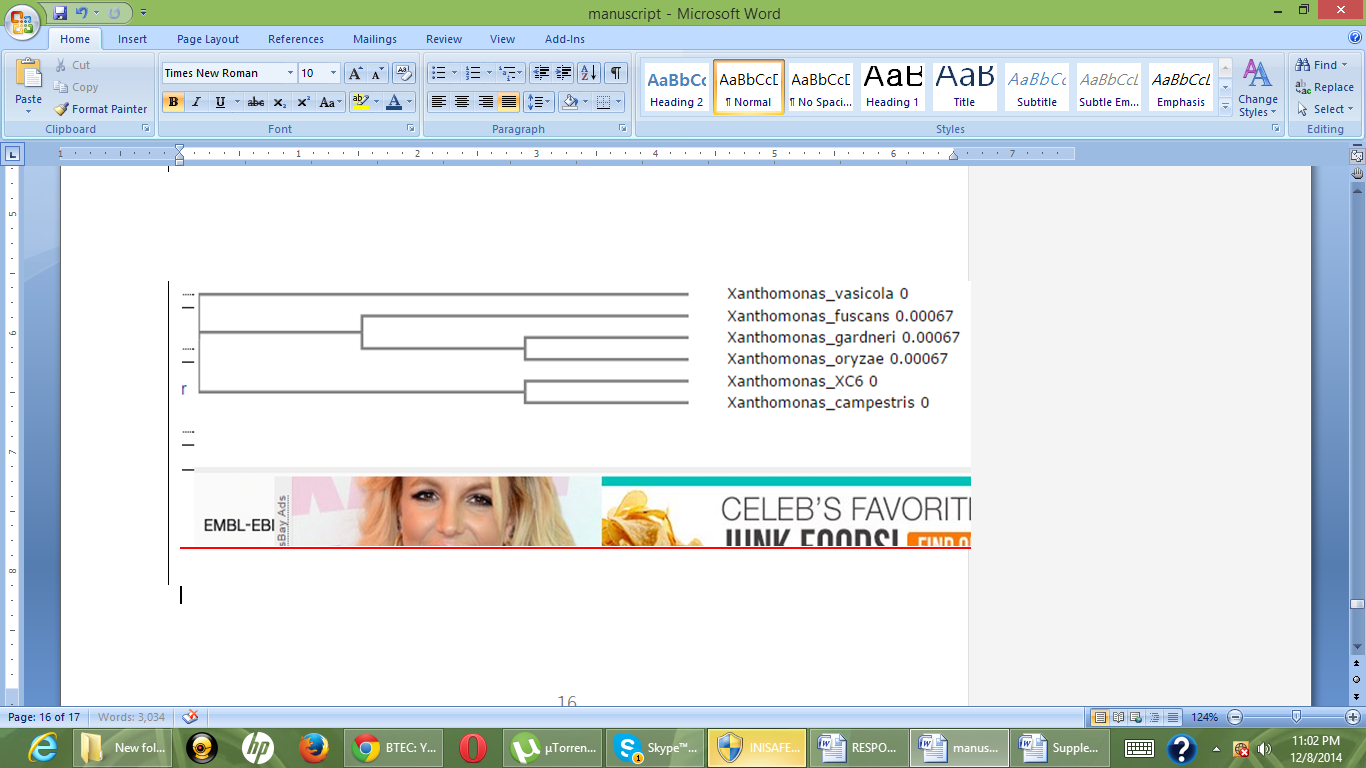
**

# Figure S2 Xanthan gum production profile of *Xanthomonas* sp. XC6 on different nitrogen source

**Table S1. Phenotypic and biochemical characterization of isolated bacterial strain XC6**

| **S.No** | **Phenotypic and Biochemical character** | | ***Xanthomonas* sp. XC6** |
| --- | --- | --- | --- |
| 1 | Morphology | | Rod |
| 2 | Color | | Yellow |
| 3 | Gram staining | | Negative |
| 4 | Motility | | + |
| 5 | Gelatin hydrolysis | | + |
| 7 | Starch hydrolysis | | + |
| 8 | Catalase | | + |
| 9 | Xanthan gum production | | + |
| 10 | Carbon utilization | Glucose | + |
| 11 |  | Fructose | + |
| 12 |  | Sucrose | + |
| 13 |  | Maltose | + |
| 14 |  | Glycerol | - |
| + = Indicates positive result  - = Indicates negative result | | | |
